# Application of machine learning techniques for creating urban microbial fingerprints

**DOI:** 10.1186/s13062-019-0245-x

**Published:** 2019-08-16

**Authors:** Feargal Joseph Ryan

**Affiliations:** 1 APC Microbiome Ireland, University College Cork, Cork, Ireland; 2 South Australian Health and Medical Research Institute, Adelaide, Australia; 3 Flinders University, Adelaide, Australia

**Keywords:** Microbiome, Machine learning, Public health, Urban, Bioinformatics, Microbiota

## Abstract

**Background:**

Research has found that human associated microbial communities play a role in homeostasis and the disruption of these communities may be important in an array of medical conditions. However outside of the human body many of these communities remain poorly studied. The Metagenomics and Metadesign of the Subways and Urban Biomes (MetaSUB) International Consortium is characterizing the microbiomes of urban environments with the aim to improve design of mass transit systems. As part of the CAMDA 2018 MetaSUB Forensics Challenge 311 city microbiome samples were provided to create urban microbial fingerprints, as well as a further 3 mystery datasets for validation.

**Results:**

MetaSUB samples were clustered using t-SNE in an unsupervised fashion to almost discrete groups, which upon inspection represented city of origin. Based on this clustering, geographically close metropolitan areas appear to display similar microbial profiles such as those of Auckland and Hamilton. Mystery unlabeled samples were provided part of the challenge. A random forest classifier built on the initial dataset of 311 samples was capable of correctly classifying 83.3% of the mystery samples to their city of origin. Random Forest analyses also identified features with the highest discriminatory power, ranking bacterial species such as *Campylobacter jejuni* and *Staphylococcus argenteus* as highly predictive of city of origin. The surface from which the sample was collected displayed little detectable impact on the microbial profiles in the data generated here. The proportion of reads classified per sample varied greatly and so de-novo assembly was applied to recover genomic fragments representing organisms not captured in reference databases.

**Conclusions:**

Current methods can differentiate urban microbiome profiles from each other with relative ease. De-novo assembly indicated that the MetaSUB metagenomic data contains adequate depth to recover metagenomic assembled genomes and that current databases are not sufficient to fully characterize urban microbiomes. Profiles found here indicate there may be a relationship between geographical distance between areas and the urban microbiome composition although this will need further research. The impact of these different profiles on public health is currently unknown but the MetaSUB consortium is uniquely suited to evaluate these and provide a roadmap for the inclusion of urban microbiome information for city planning and public health policy.

**Reviewers:**

This article was reviewed by Dimitar Vassilev, Eran Elhaik and Chengsheng Zhu.

**Electronic supplementary material:**

The online version of this article (10.1186/s13062-019-0245-x) contains supplementary material, which is available to authorized users.

## Background

Microbiome research has been an area of growing interest in recent years, especially within the context of human health and disease [1]. This has found that virtually every surface surrounding humans contains a microbial community, often largely composed of uncultured microbial life often referred to as “Microbial Dark matter” [2]. Historically, microbial studies tended to focus on disease causing organisms or those important for food production with their taxonomy most often described by their physical characteristics [3]. More recently, bacterial taxonomy has relied on the 16S rRNA gene, although this approach is limited by the taxonomic resolution of this gene, which has varying levels of identity across different phyla [4]. As a result of whole genome sequencing becoming more affordable there are now tens of thousands of genomes available, which has resulted in substantial revisions to prokaryotic and viral taxonomy [5]. Many diseases have been demonstrated to be associated with alterations in the human microbiome [6] and it has been shown that there is overlap between the human microbiome and the microbiome of particular rooms with some evidence suggesting that microorganisms from these environments can colonise humans [7]. Thus, urban microbiomes may play a role in shaping the bacteria, archaea, viruses and microbial eukaryotes in our bodies and may play a role in health. The Metagenomics and Metadesign of the Subways and Urban Biomes (MetaSUB) International Consortium aims to characterise the microbiome of mass transit systems and cities from around the world [8]. This work found that the identifiable organisms contained bacterial genera associated with human skin but that approximately 50% of sequences generated matched no known organism [8].

Here, we report on an analysis of the CAMDA 2018 MetaSUB Forensics Challenge dataset which supplied 393 city microbiome profiles with the aim of constructing urban microbiome fingerprints and find the geographical origin of mystery samples. Samples were classified against the NCBI nr database with Kaiju [9] a taxonomic classifier which performs 6 frame translation which aids in the detection of distant homologous relationships. Utilizing t-Distributed Stochastic Neighbor Embedding (t-SNE) [10] for dimensional reduction and random forest for classification and feature selection [11] it was shown that it is possible to distinguish between cities by metagenomic sequence alone.

## Materials and methods

The quality of the raw reads was visualized with FastQC v0.11.3 [12] followed by read trimming and filtering with Trimmomatic v0.36 [13] to ensure a minimum length of 60, maximum length of 100, and a sliding window that cuts a read once the average quality in a window size of 4 falls below a Phred score of 30. Sequence reads were classified into known taxonomic groups using the Kaiju metagenomic classifier [9] and the NCBI non-redundant protein database as of February 6th 2018. During database construction Kaiju uses a list of NCBI taxonomic identifiers to determine which taxa are included in the database for indexing which was altered here to include sequence from all domains of life rather than just bacteria. Following classification, per read counts of each taxonomic rank per sample was generated for use in further analysis. Quality filtered reads were assembled per sample with the MegaHIT assembler [14]. Random forest [11], t-SNE based on Spearman distance between samples [10] and visualization was performed in R v3.3.0. The random forest classification implemented here was done on the default parameters with 500 trees. Feature importance was then extracted from this model to rank features by their contribution to the model. A recursive feature eliminated step was implemented removing the 20% of features of least importance on each iteration (as judged by mean decrease in accuracy) for 100 iterations. All plots were generated using ggplot2 [15]. All R code has been provided as per the data availability statement below.

## Results

The initial CAMDA challenge dataset consisted of 311 samples from 8 cities across 6 countries (Table 1). Samples from New York (NY) and Sacramento could be further broken down to those sequenced as part of a pilot and a later study (labelled as csd2016). The CAMDA 2018 data included a further 82 “mystery samples” as part of 3 challenges. Challenge 1 (C1) samples were from cities previously featured in the dataset but are unlabeled, Challenge 2 (C2) samples were from 3 cities not previously featured and marked as City 1, 2 and 3. Challenge 3 (C3) samples were a mix of new and those previously featured in the dataset with no information about which belong to the same city (Additional file 4: Table S1).

**Table 1:** Description of MetaSUB challenge dataset

City	Country	Number of samples
Auckland	New Zealand	15
Hamilton	New Zealand	16
New York	U.S.A.	126
Ofa	Nigeria	20
Porto	Portugal	60
Sacramento	U.S.A.	34
Santiago	Chile	20
Tokyo	Japan	20

### MetaSUB microbiome composition and unsupervised clustering

**Fig. 1:**
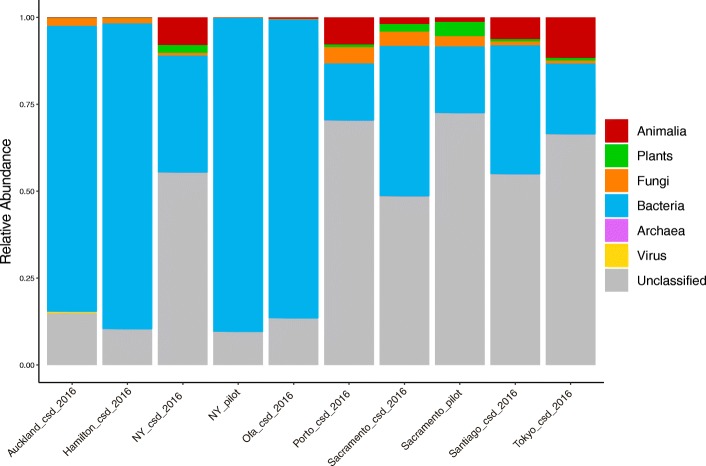
Barplots of relative abundance for domains of life per city in the MetaSUB challenge dataset

**Fig. 2:**
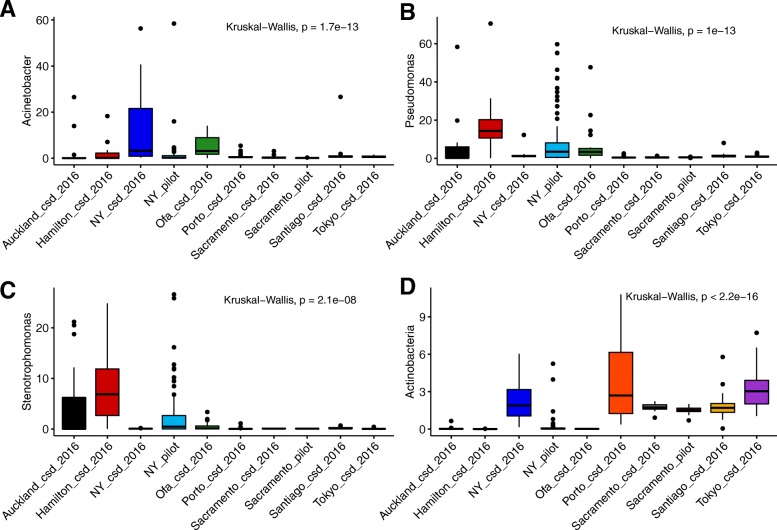
Boxplots of relative abundance of most abundant taxa in the primary CAMDA dataset of 311 samples. Relative abundance of **a** Acinetobacter, **b** Pseudomonas, **c** Stenotrophomonas and **d** Actinobacteria. Kruskal Wallis *P* values are represented on each plot

**Fig. 3:**
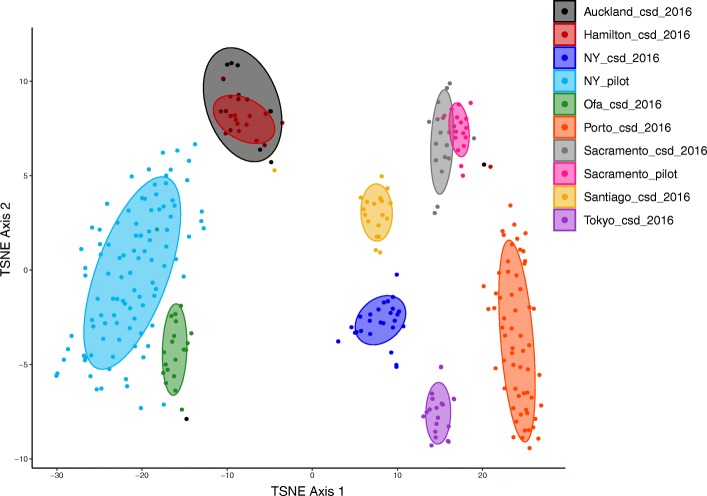
t-SNE output to represent microbial profiles on two dimensions. Spearman dissimilarities were calculated from a set of 2239 taxonomic features which represent those present in at least 5% of samples with a minimum relative abundance of 0.1% in a single sample. Confidence regions are 70% confidence regions showing surface type

As it was previously reported that a large percentage of the sequences from MetaSUB matched no known organism [8] a translated search method, Kaiju, was utilized to examine these data as searching in amino acid space allows for the detection of more distant homology [9]. In order to provide an overview of total sample composition, a Kaiju database was constructed from the NCBI nr database containing sequences from Animalia, Plants, Bacteria, Archaea, Viruses and Fungi (Fig. 1). The amount of sequence classifiable to any domain of life varied considerably from as low as less than 1% to over 80%. This approach found that the amount of DNA classified as Animalia varied largely between cities, as did the total amount of sequence which was classifiable. In all cases, the majority of identifiable sequence corresponded to Bacteria. The most abundant genera detected throughout the dataset were *Pseudomonas*, *Acinetobacter* and *Stenotrophomonas* (Fig. 2a-c, Additional file 5: Table S2) all members of the *Gammaproteobacteria*. Aside from this, *Actinobacteria* was the most abundant phylum throughout the data (Fig. 2d, Additional file 5: Table S2). All of these taxa show highly significant differences by city when assessed by Kruskal Wallis test (Fig. 2). In those cities which displayed higher amounts of sequence from the domain Animalia this was due to DNA classified as the phylum *Chordata*, within which it was primarily belonging to the genus *Homo*. Sequences corresponding to fungi and other microbial eukaryotes such as the *Tardigrada* and the *Mollusca* were also detected. In this analysis we focused primarily on sequences classified as Bacterial, but the importance of non-Bacterial microorganisms has been noted in the context of other microbiomes [16]. A full list of all detected taxa is available via the supplementary data (Additional file 6: Table S3). A microbial count table was generated by taking only counts of sequences classified to any rank from Bacteria, Archaea, Fungi, microbial eukaryotes or Viruses only. For example, the Domain Bacteria, the Phylum *Proteobacteria* and Class *Gammaproteobacteria* were all present as distinct features, where the counts of the Bacteria represented the reads which could only be classified as far as Domain, the counts of the *Proteobacteria* represent the number of reads per sample which could be classified at the phylum level and so on. This approach was implemented to utilize the maximum amount of information per sample as it allows for the inclusion of the amount of unclassified sequence as a feature. This resulted in a table of 311 samples with 75,648 features. Uninformative features were removed by filtering for those which were present in at least 5% of samples with a minimum of 0.1% relative abundance in any one sample which resulted in 2239 features (Additional file 6: Table S3). This subset of feature counts was then used as input to t-SNE for unsupervised dimensional reduction (to 2 dimensions) and visualization (Fig. 3) This approach demonstrates that urban microbial profiles largely cluster in an unsupervised manner by city of origin except for Auckland and Hamilton which appear indistinguishable. This also shows the large differences in the New York samples between CSD 2016 and the pilot samples, although Sacramento samples cluster together regardless of dataset (Fig. 3).

### Random forest classification and feature importance ranking

In order to identify features which were key to discriminating cities, random forest was used to classify samples into their city of origin and rank features by importance to the model. A certain level of correlation between features was expected for these data for several reasons. Genomic sequence shows variation in the level of conservation (and thus the ability to classify sequence to lower taxonomic ranks) which may cause correlation between these features. Microorganisms may also show co-abundance relationships also leading to highly correlated features. As correlated features have been found to impact the ability of random forest to identify strong predictors, a recursive feature eliminated step was implemented [17], removing the 20% of features of least importance on each iteration (as judged by mean decrease in accuracy) for 100 iterations. Using this method, it was possible to achieve a classification accuracy of 95.18% with 587 features (Table 2) with the erroneous classifications in large part due to Auckland and Hamilton, in agreement with the results from t-SNE (Fig. 2). Although this high classification accuracy is very likely due to overfitting it does allow for ranking features which discriminate between cities. *Campylobacter jejuni* was found to be most important feature by metric, followed by *Staphylococcus argenteus* (Additional file 7: Table S4, Additional file 1: Figure S1). Interestingly, both bacteria are relevant in human health.

**Table 2:** Confusion matrix showing number of correct and incorrect classifications per city from random forest analysis

	Auckland	Hamilton	NY	Ofa	Porto	Sacramento	Santiago	Tokyo	class.error
Auckland	9	5	0	0	1	0	0	0	0.4
Hamilton	4	12	0	0	0	0	0	0	0.25
NY	0	0	124	1	0	1	0	0	0.01587302
Ofa	0	0	0	20	0	0	0	0	0
Porto	0	0	0	0	60	0	0	0	0
Sacramento	0	0	0	0	1	33	0	0	0.02941176
Santiago	1	0	1	0	0	0	18	0	0.1
Tokyo	0	0	0	0	0	0	0	20	0

### CAMDA MetaSUB forensics challenge

**Fig. 4:**
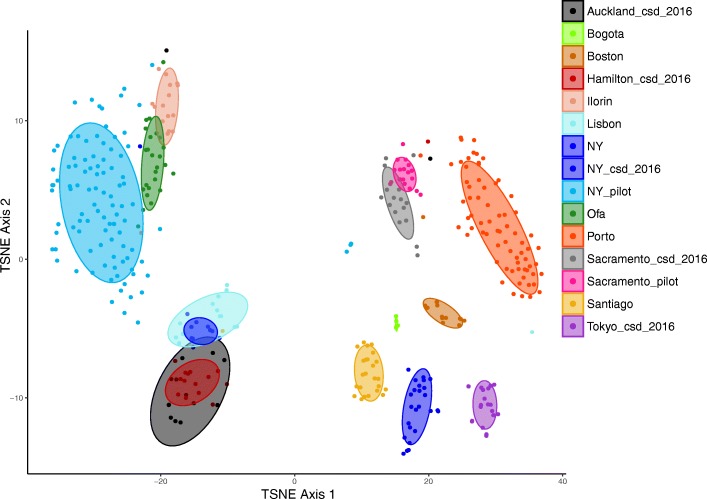
t-SNE output to represent microbial profiles on two dimensions. Spearman dissimilarities were calculated from a set of 2463 taxonomic features which represent those present in at least 5% of samples with a minimum relative abundance of 0.1% in a single sample. This includes “mystery” samples which were initially unlabeled in the MetaSUB challenge. Confidence regions are 70% confidence regions showing surface type. Samples labelled as NY are those which were marked as New York but information was not provided on which of the sample sets (csd2016 or pilot)

As part of the CAMDA challenge unlabeled samples were provided which represented cities previously included in the 311 sample primary dataset. Additional file 2: Figure S2 demonstrates the results of the C1 classification, showing where each mystery C1 sample clusters in an unsupervised fashion. Of the 30 samples in the C1 challenge, a random forest model trained on the initial 311 samples was able to correctly classify 25 of the 30 (Additional file 8: Table S5). Oddly, samples labelled as NY (indicating New York) in mystery challenge C1 clustered with New Zealand in all analyses. It was not provided if these samples were from the CSD_2016 or pilot sample collection. As mentioned above several cities were initially introduced as mystery cities, with the labels revealed following analysis. Along with samples from cities already featured in the initial 311 sample dataset, there was samples from a further 4 cities added – Bogota, Boston, Ilorin and Lisbon. Repeating the t-SNE analysis with this dataset of 393 samples highlighted largely the same pattern that urban microbial profiles cluster by city of origin in an unsupervised fashion (Fig. 4). Like Auckland and Hamilton, the nearby urban areas of Ofa and Ilorin cluster together based on this analysis potentially indicating intra-country signals. As noted above the city of origin had a large impact on microbial profile, thus in order to investigate the impact of collection surface the dataset was reduced to only those samples from New York, which contained more samples and sample types than any other city featured in this dataset. Within the New York data, microbial profiles as generated here were unable to resolve surface type across different cities (Additional file 3: Figure S3).

### De novo metagenomic assembly

As noted above, and in previous analyses of urban microbiomes, large portions of the sequences are not assignable to any taxonomic group [8]. In order to investigate microbial sequences not represented in databases and the viability of using the MetaSUB data for assembling novel genomes, each sample was assembled with Megahit, an assembler designed for large and complex metagenomics data [14]. When filtered for a minimum length of 5000 bases, this generated 183,100 assemblies, 5502 of which were over 100,000 bases. The largest sequence assembled was 1,772,995 nucleotides long, from a sample from the city of Hamilton. Homology searches in the nt nucleotide database at NCBI showed this contig to share regions of 5 – 10 kb at approximately 90% identity with members of the family *Enterobacteriaceae* indicating this may represent a species not well covered in reference databases.

## Discussion

The random forest and t-SNE approaches implemented here represents a relatively simple approach which in some cases only classifies a small percentage of reads, but even with this it is often possible to correctly classify the sample by city of origin. The t-SNE based analysis indicated that nearby urban environments such as Auckland and Hamilton and Ofa and Ilorin have similar microbial profiles. This may indicate a relationship between geographical distance between cities and similarity of their microbiomes. As human populations have been found to impact these microbial communities this may be due to movement of people between areas. As the MetaSUB consortium represents the first concerted effort to characterize urban biomes there is little other studies which can act as a point of comparison. *Campylobacter jejuni*, which we find here as increased in Porto and Sacramento is relevant in food contamination, has been found to persist on surfaces in a poultry processing facility for up to 120 min lending some credence to the findings here [18]. *Staphylococcus argenteus*, a member of the *Staphylococcus aureus* complex which may cause skin infections [19], follows a similar pattern of abundance to *C. jejuni*. Sequences classified as fungi and as members of the cyanobacteria are also ranked as important to discriminating between cities. Many of the taxa which we find as most abundant are not those commonly found on human skin, such as *Acinetobacter* and *Pseudomonas* [20]. However, these are frequently isolated from environmental sources indicating that the composition of these metagenomes is a mix of environmental and human association microorganisms. The taxa mentioned here as identified in this study have not been confirmed via any laboratory test, and thus may be subject to the accuracy limitations of any metagenomic classification approach. Importantly non-human microbiomes are underrepresented in reference databases [21] and so taxa from these environments may be more prone to misclassifications. The ability to correctly identify the majority of the C1 mystery dataset does indicate that city specific microbial signatures may exist and warrants further study. When interpreting these results, it is important to note that there is no temporal component to the sample collection in this study. There are samples from New York which were collected and sequenced at different times and that show different microbial profiles. This may be indicative of differences by season, weather or perhaps some batch effect from differential processing techniques. This is potentially a major limitation in identifying biomarkers of an urban biome as when climate, weather and season are considered large intra-city variations in the microbiome may be observed. Research on microbial communities in the environment has found changes associated with seasons [22, 23]. Human contact has been shown to contribute to the microbial sequences observed in MetaSUB and so seasonal differences in clothing may also shape these communities [8]. The previous analysis of urban biomes by Afshinnekoo et al. found an enrichment of bacteria associated with the skin potentially indicating that the human population are one of the majority sources of variation between environments and so frequency or duration of human skin contact may be an important factor [8].

### Urban microbiome sequence classification & identification

Kaiju is a metagenomic classifier based on amino acid homology and was chosen here as amino acid homology may allow for the detection of distantly related sequences as the initial MetaSUB dataset indicated large amounts of unclassified sequence [8]. Here we have not conducted robust testing of the bioinformatics methodology relying on published reports of accuracy and have instead focused more simply on if it is possible to between urban areas based on microbiota composition. Such benchmarking would at the minimum involve testing a variety of databases and algorithms, covering both nucleotide and amino acid homology and reference-based vs de novo approaches. This would be the logical next step in establishing a classification approach for both the MetaSUB dataset. However, the results presented here provide strong evidence that such an effort would be successful in establishing a robust and accurate microbial fingerprinting method for urban biomes. The choice of reference database for any classification approach is a key consideration and can have a large impact on results and analysis [24]. Here the nr database from NCBI was chosen for several reasons, primarily that a large resource of protein sequences. Amino acid homology was prioritized here as previous work in MetaSUB generated metagenome has indicated a large amount of uncharacterized sequences [8] and this would allow for the detection of more distant homology. The nr database is also well known in the field and thus would be familiar and easily available to other users seeking to reproduce this work. While the large size of the database is beneficial in classifying more sequences it also may be problematic for users with limited computational resources as a large amount of RAM is required is indexing. The nr database is also not version tracked which may be an issue for version control (The date on which the database was downloaded was used as a proxy here). Many other resources could be leveraged to create a bespoke database that could provide more information on the microbial life contained within these urban biomes. For example, Pasolli et al. have recently metagenomic assembly of over 9000 metagenomes and demonstrated the utility of metagenomic assembly for expanding our knowledge about the microbial world [25]. A similar approach incorporating human associated and environmental metagenomes which together with curation could provide an excellent resource for studying urban biomes. As previously described there are a large number of uncharacterized and uncultured bacteria and viruses present in the MetaSUB metagenomic data [8] and here we confirmed this by performing an assembly based analysis in concert with classification. Successful assembly of the sequence data from the MetaSUB project indicates that it is possible to mine for novel genomes which can further capture variation in these environments as has previously been done for the human microbiome [25, 26].

### Considerations for future studies of urban microbiomes

This study represents an initial attempt to establish to what degree the urban microbiome can distinguish between cities, countries and surface types. While the results here are promising there remains several important considerations that warrant further investigation. Specifically, the choice of reference database as mentioned above, and the choice of classification technique and dimensional reduction techniques which were not bench marked here. Random forest was implemented here as it represents one of the simplest and most widely used techniques in microbiome research for classification and thus will be familiar and easily implemented by researchers seeking to reproduce this methodology. While overfitting is always a concern with classification, we do not believe it to be a major impact on the results presented here due to the large sample size, t-SNE clustering results, and clear differences by cities in the abundance of multiple taxa. Although not applied here, the MetaSUB data also represents an excellent opportunity to apply geospatial and leverage microbiome data for phylogeography analysis – that is relate phylogeny to spatial and environmental factors [27]. Furthermore, it would be interesting to utilize information about the city latitude, climate, type of transit system, number of passengers, ambient temperature and other data to further identify what differences, if any, exist intra-city as compared to inter-city.

## Conclusions

This work has shown that with current databases and methods it is possible to create a microbial fingerprint for cities and urban areas from across the world. Geographically close urban environments such as Auckland and Hamilton are shown to have similar microbiome profiles. A large portion of the sequence in the MetaSUB dataset is not classifiable and so future analyses of urban biomes would benefit from mining for novel genomes, and extensive exploration of the uncultured microbiome as has been done for the human microbiome. Although the impact of these communities on the public health and wellbeing is yet undescribed, the MetaSUB consortium contains the potential to impact both urban planning and public health policy in the future.

## Reviewers comments

### Reviewer 1 report 1- Dimitar Vassilev

1. The use of the NCBI nr data base. Is it the only information resource for classification of the samples. Is it possible to use some other external information sources - which can add some knowledge to the obtained results?

Author Response: *It is the only database used here but that was primarily because we wanted to implement an approach that was as simple and reproducible as possible. Yes, it is possible to include other information sources and we have amended the manuscript to include further discussion [lines 226 to 240].*

2) The classification methodology. At first side everything looks like in a well known recipe. Are there some related works which can confirm or reject the authors approach. How we can evaluate the authors approach?

Author Response: *The methods are based off a description of benchmarking of the Kaiju classifier with the nr database in the original Nature Communications Kaiju publication. However, bench marking such an approach is key. But in order to do this adequately it would require a comparison of multiple databases, classification approaches and assembly-based methods that we saw as beyond the scope of this particular challenge as the goal of the CAMDA challenge was to identify if it was possible distinguish between cities using microbial fingerprints. We believe the next step is to establish which method and reference database would be best. We have included this in the discussion at lines 218 to 225.*

3) The Machine learning models: Random forest is widely used for research – because of its power and decent accuracy, and performance. However, the major problems of random forest is the unbalanced data, low interpretability, problems with overfitting and selection parameters. Random forest is used when you are just looking for high performance with less need for interpretation. In this line, can author give some more reasons for using particularly only the RF and could be applied another machine learning models. This can be regarded as a sort of the validation of the presented approach and the obtained results. In data science applied to biology there is always a sharp need for validation of the results.

Author Response: *A very important point. As mentioned above the goal of this study was to assess the viability to use urban microbiomes to distinguish cities rather than evaluate and benchmark all potential approaches. Thus, differences in particular taxa were highlighted, and unsupervised clustering was implemented. Future work will absolutely have to address this question. For this initial evaluation we wanted to use an approach that would be as simple and reproducible as possible. While overfitting is a major concern in classification there are a combination of things which we believe indicate that it is not a concern here. First the large sample size, second large separation between groups observed in t-SNE plots and third that the features identified by random forest as important are clearly very different between cities. This has been discussed at lines 251 to 255.*

4) The geographical classification can be regarded as another issue for potential methodological extension. The t-SNE approach is necessary to be validated also: there is a large choice of unsupervised machine learning models as well as the opportunities of the Geo Spatial approaches.

Author Response: *Again, we agree with the reviewer on this point and have added to the main text (in the same section as point 3 above) that a robust comparison and evaluation of all methods is the necessary next step now that we have established there appears to be a strong microbial signal that distinguish cities. We had initially planned to include some geo spatial analytical approaches but unfortunately was not able to due to time considerations*.

5) Finally, the style of the submitted material. It looks more as a report of the project. We hope the author can make his best efforts to present the material in a more paper-like shape. Regardless of the criticisms and the remarks we have, we would recommend to the editors of the issue to suggest the submitted material for publication after major revision.

Author response: *This has been corrected throughout to reflect a more publication style format following the submission guidelines of BMC Biology Direct.*

### Reviewer 2 report 1- Eran Elhaik

1. Page 7, lines 44–50. Where are the classification results for these 4 cities?

Author Response: *This is based on clustering by t-SNE analysis. The text has been amended to state this clearly.*

2) Overall, I am missing the classification results for C2 and C3. Results should clearly say which dataset is being analyzed.

Author Response: *The full list of all samples, which city and challenge they are from is listed in Supplementary Table 1 and in the results section.*

3) The point of the challenge was to use C1 to train the classifier and demonstrate the accuracy on C2 and C3. These results are not reported. We appreciate that they may not be very good, but they have to be reported nonetheless, so that we would know how to evaluate the classifier.

Author Response: *That was not the point of the challenge. There was a primary dataset which contained 311 samples from locations that was disclosed and three challenge datasets C1, C2 and C3 with unlabeled samples. It was never the intention of the challenge to use C1 to classify others. C1 (30 samples) was where the location was unknown, but the location was already in the primary dataset, but both C2 (36 samples) and C3 (16 samples) contained cities/countries not featured in any other dataset. Thus, one could not train on the original samples or train on C1 and assess performance on C2/C3. This can be seen in the supplementary data we have provided. However, in order to answer this question, we have provided a supplementary figure which shoes how the unlabeled C1 samples cluster with the primary dataset of 311 samples.*

4) There should be more discussion about Fig. 3. Can you explain these results? You should establish whether they are supported in the literature or not? If not, then these are not good forensic biomarkers and may be due to chance/season/some other temporary event. This is not a negative finding, but it needs to be properly reported. People should know whether these findings can be expected to be replicated.

Author Response*: Further discussion has been added (Lines 195–210) but given the novelty of the MetaSUB data, it’s not possible to verify all of these findings in the literature. To my knowledge no other study has examined urban microbiomes in this fashion.*

5) Page 8, 14–36. What is the purpose of this analysis? Why is it here? Shouldn’t it be at the beginning since it evaluates the data?

Author Response: *The purpose of this analysis is stated in the text. It was to indicate the benefits of leveraging de-novo. Several large studies have been published in Nature and other high impact journals demonstrating the utility of such approaches in the human microbiome.*

Minor issues

i.) The term “microbes” is not a scientific one. Bacteria or microbiome are better.

Author Response: *Language has been changed throughout to be more accurate.*

ii.) All R packages should be referenced.

Author Response: *All packages are now referenced.*

iii.) Page 6, line 22, “Other detected members” what other? Avoid using ambiguous terms like this.

Author Response: *The language in this sentence has been changed to clarify. The full list of taxa detected with this approach they are available in the supplementary material*.

iv.) Page 8, Afshinnekoo et al. – add citation.

Author Response: *This citation has been added.*

v.) Page 9, “This highlights the large challenge facing” - > challenges.

Author Response: *This typo has been corrected.*

vi.) Why no figure/table legends. Please put some effort into explaining the table/figures better.

Author Response: we *have included expanded legends and put them in the main text document. I’m unsure why they weren’t made available to the reviewers previously.*

vii.) Typos.

Author Response: *These have been corrected.*

### Reviewer 2 report 2 - Eran Elhaik

1. There are unclear sentences and punctuation signs are missing.

Author response*: The manuscript has now been corrected.*

2) Line #195 how did you get to 587 features from 2239 features reported in line #179?

Author Response: *The number of features was reduced by recursively removing features of lowest importance. The manuscript has been altered to state this in a clearer fashion. The R code used for this analysis is available per the data availability statement.*

3) Please do the following state clearly that you developed a classification, not a prediction algorithm & report the accuracy of the algorithm on the C1 dataset. This would provide a fair evaluation of the classification accuracy of your algorithm.

Author Response: *The manuscript has been changed to state clearly it is a classification and the accuracy on the C1 dataset is stated in the abstract, results section and a supplementary data.*

Minor issues

i) *Poor grammar, line #28: “microbial communities both in and surrounding human”.*

Author response: *This has been corrected.*

ii) *This sentence makes no sense: “As part of the CAMDA 2018 MetaSUB Forensics Challenge hundreds of city microbiome samples were provided to create urban microbial fingerprints.”*

Author response: *This has been corrected.*

iii) *Line #37 - > geographical - > geographically Data are plural.*

Author response: *This has been corrected.*

iv) *Line #54 and #79: “Eran Elhaikand” - > “Eran Elhaik”.*

Author response: *Apologies for the typo. This has now been corrected.*

v) *Lines #104–105: “However”? where is the contradiction?*

Author response: *The language has been corrected in this section.*

vi) *Line #119 – who are “they”?*

Author response: *The language has been clarified in this section.*

vii) *Line #122 – “I report on results of the CAMDA 2018 MetaSUB Forensics Challenge” – clarify, it sounds like you cover the challenge.*

Author response: *This has been clarified.*

viii) *Line #123 – “hundreds of novel city microbiome profiles” – can you be more precise?*

Author response: *The precise number of samples is now stated.*

ix) *Line #170, missing period. Also, which “supplementary data”? doesn’t it have a name? which microbial count table?*

Author response: *The exact supplementary data in reference is now stated.*

x) *Line #186 makes no sense.*

Author response: *It has been clarified.*

### Reviewer 3 report 1 - Chengsheng Zhu

Some statements in this paper would be clearer if the author could offer more details, especially in the machine leaning part.

1. It is not clear in text what the taxa features exactly represent. The author described the feature “Domain Bacteria” represents “the reads which could only be classified as far as Domain” (page 5 line 35). To me this means that reads that can be classified to lower taxonomic rank features, e.g. “Proteobacteria”, are not included in higher taxonomic rank features, e.g. “Domain Bacteria”. In this notion, all the taxa features are actually exclusive, i.e. one read can only be counted in one feature. It thus confuses me when the author later describes, “…Due to the nature of how the counts were generated highly correlated and related features may be present in the data such as Proteobacteria and Gammaproteobacteria…” (page 6 line 11). Based on the above, we don’t see how “Proteobacteria” and “Gammaproteobacteria” are correlated and related in this case. More clarification can be helpful.

Author Response: *Reviewer 3 is correct in their assessment that the counts are exclusive, however a high level of correlation is still seen. This we believe is primarily because not all sequence is a genome can be classified equally well to a taxonomic rank, some areas will be more conserved or variable. In the dataset here, we see a modest correlation between Proteobacteria and Gammaproteobacteria for example (Spearman’s rho 0.66). It is also possible that co-abundant groups of taxa are present here similar to those described in other biomes. However, reviewer 3 makes a good point and so we have further clarified this in the text (Lines 142 to 147).*

2) The author reported his Random Forest model reaches over 95% accuracy in predicting samples’ city origin. It is not mentioned in text how the author deals with potential overfitting, i.e. what are the parameters of the random forest run? Did the author do a manual Cross-Validation? In addition, we would also suggest the author report the model’s performance on C1 set for more thorough evaluation.

Author Response: *Thank you for the comment. We feel a better explanation of the logic behind the choice of random forest for this analysis would be beneficial here as we now see it was not apparent in my previous draft. We utilized Random Forest primarily to report a classification accuracy (as it was obvious from t-SNE that such a method should be able to classify these with ease) and then rank important features. We reported the classification accuracy & confusion matrix as we assumed readers would be interested. Here, the accuracy of the random forest classification (especially in the confusion matrix) is shows nearly identical results to the unsupervised clustering shown in the t-SNE plot and thus we do not think overfitting a large concern here (Given how well many of the cities separate). We do acknowledge that it is an issue for evaluation of such methods and will be key in future work if a classification approach is utilized. The manuscript has been changed throughout to emphasize the use of random forest here as a feature selection technique primarily.*

Minor issues

i.) Page 4 line 28. It is not explicit that “counts of each taxonomic rank” means read counts.

Author Response: *This has been clarified.*

ii.) Page 5 line 26. “the highest possible taxonomic rank” is quite confusing and inaccurate.

Author Response: *This has been clarified.*

iii.) Page 6 line 22. “…a classification accuracy of over 95.82%..” This accuracy is not in line with Table 2.

Author Response: *Apologies the value represents a typo. It should have been 95.17%.*

iv.) Page 6 line 24. The statement of errors being “… almost entirely due to Auckland and Hamilton…” is not correct, as Auckland-Hamilton confusion accounts for a bit less than half (~ 2%) of the total errors (~ 4%).

Author Response: *Apologies, we should have been more precise in my language and this has been corrected.*

v.) What are the criteria to choose those four taxa in Fig. 3? If the point is merely to showcase differentially abundant taxa across the cities, we would recommend including statistic tests to make the statement stronger.

Author Response: *Those features were chosen based on the importance from random forest and were chosen to highlight that certain taxa are differential between cities. We chose not to implement a statistical test across all features as the multiple testing adjustment would be prohibitive. However, we have opted to include the most highly abundant features and full list of important random forest predictors in the supplementary to make this clearer.*

vi.) In Fig. 4, what is the “NY” in the legend?

Author Response: *Apologies, this should have been made clearer. These represent samples labelled as New York in the challenge datasets, but information was not provided on if they came from the csd_2016 data or the pilot dataset. The figure legend has been updated to reflect this and this has been mentioned in the main text (line 157).*

### Reviewer 3 report 2 - Chengsheng Zhu

1. As the author carried out random forest with all default settings, the reported 95.18% accuracy is, not potentially but definitely, overfitted – the author should make it clear in the text. On the other side, it is great that the author now includes the performance on C1 test set, which offers a more objective view on the true performance of the cluster. We suggest the author to discuss this point more thoroughly.

Author Response: *We thank the reviewer for their suggestion. We have altered the text to make it clear in the results that the 95.18% classification accuracy is very likely the result of overfitting and focus more on the C1 test set for discussion (Line 200).*

2) The description of how random forest is carried out should go to methods.

Author Response: we *have moved this text to the methods (Line 142). The full code for the entire analysis is also available as per the data availability statement.*

3) In the new t-sne figures, there are overlapping color labels.

Author Response: we *apologise for this oversight. An indexing error in R. The figure has been corrected and the colour scheme now matches that of other figures.*

4) It is good that the author expands the discussion. While we appreciate the author’s effort to perform assembly analysis as an additional component, it is a rather minor result of this manuscript – one paragraph of brief text without any figures or tables. However, a significant fraction of the discussion is dedicated to assembly, which doesn’t seem adequate and miss the point. We would suggest the authors to focus on the taxa he identified (as in Fig. 3), as this is the main point from my impression.

Author Response: *We thank the reviewer for their suggestion and adjusted the manuscript. We have reduced the text dedicated to the assembly analysis in the results and conclusions (lines 221 to 229 & 292 to 295) and increased the text related to the identified taxa (lines 164 to 170, 201 to 204, 239 to 252). However, we are hesitant to overinterpret the results of the classification of any particular species due to lack of further confirmation with culture/lab-based testing. In my opinion the key finding here is that it is possible to distinguish between cities using current reference databases, but that until better reference databases are available urban microbiome metagenomic fingerprinting would benefit from inclusion of a* de novo *reference database.*

## Additional files

Additional file 1: **Figure S1.** Relative abundance profiles of taxa identified as Random Forest as most important in distinguishing between cities. (PDF 88 kb)

Additional file 2: **Figure S2.** t-SNE output to represent microbial profiles on two dimensions. Spearman dissimilarities were calculated from a set of 2347 taxonomic features which represent those present in at least 5% of samples with a minimum relative abundance of 0.1% in a single sample. Confidence regions are 70% confidence regions showing surface type. Size and shape of points indicates those which were part of the initial 311 sample set or those which were unlabeled. Information about city of origin was not used to generate these data and thus this highlights the ability to cluster samples by city of origin. (PDF 121 kb)

Additional file 3: **Figure S3.** t-SNE output to represent microbial profiles on two dimensions. Spearman dissimilarities were calculated from a set of 2239 taxonomic features which represent those present in at least 5% of samples with a minimum relative abundance of 0.1% in a single sample. Confidence regions are 70% confidence regions showing surface type. (PDF 85 kb)

Additional file 4: **Table S1.** CAMDA Challenge mystery samples. The complete list of all samples included in the CAMDA challenge, which challenge they were released with, and their city of origin. (XLSX 10 kb)

Additional file 5: **Table S2.** Mean relative abundance of the top 100 most abundant count features throughout the 311 samples in the primary dataset. (XLSX 11 kb)

Additional file 6: **Table S3.** Count matrix of 2463 features which were present in at least 5% of samples with a minimum relative abundance of 0.1% in a single sample. (XLSX 8761 kb)

Additional file 7: **Table S4.** Importance table generated by Random Forest showing mean decrease in accuracy and mean disease in Gini associated with each feature in the random forest model. (XLSX 65 kb)

Additional file 8: **Table S5.** Random Forest assignments for each of C1 mystery challenge samples. Random forest model trained on 311 samples and then city predicted with the predict function in R. (XLSX 11 kb)

## Data Availability

All R code, along with count data generated, associated meta data and assembled contigs with a length of greater than 100 kilobases have been deposited in FigShare and are available at https://figshare.com/s/5dfede00a4f07be1cc10.
