# Supplementary material for: Application of machine learning techniques for creating urban microbial fingerprints
Source: Biol Direct. 2019 Aug 16;14:13. doi: 10.1186/s13062-019-0245-x (PMC6697990; doi:10.1186/s13062-019-0245-x)
Supplement: Supplementary file 1 — Figure S1. Relative abundance profiles of taxa identified as Random Forest as most important in distinguishing between cities. (PDF 88 kb) [file 13062_2019_245_MOESM1_ESM.pdf]

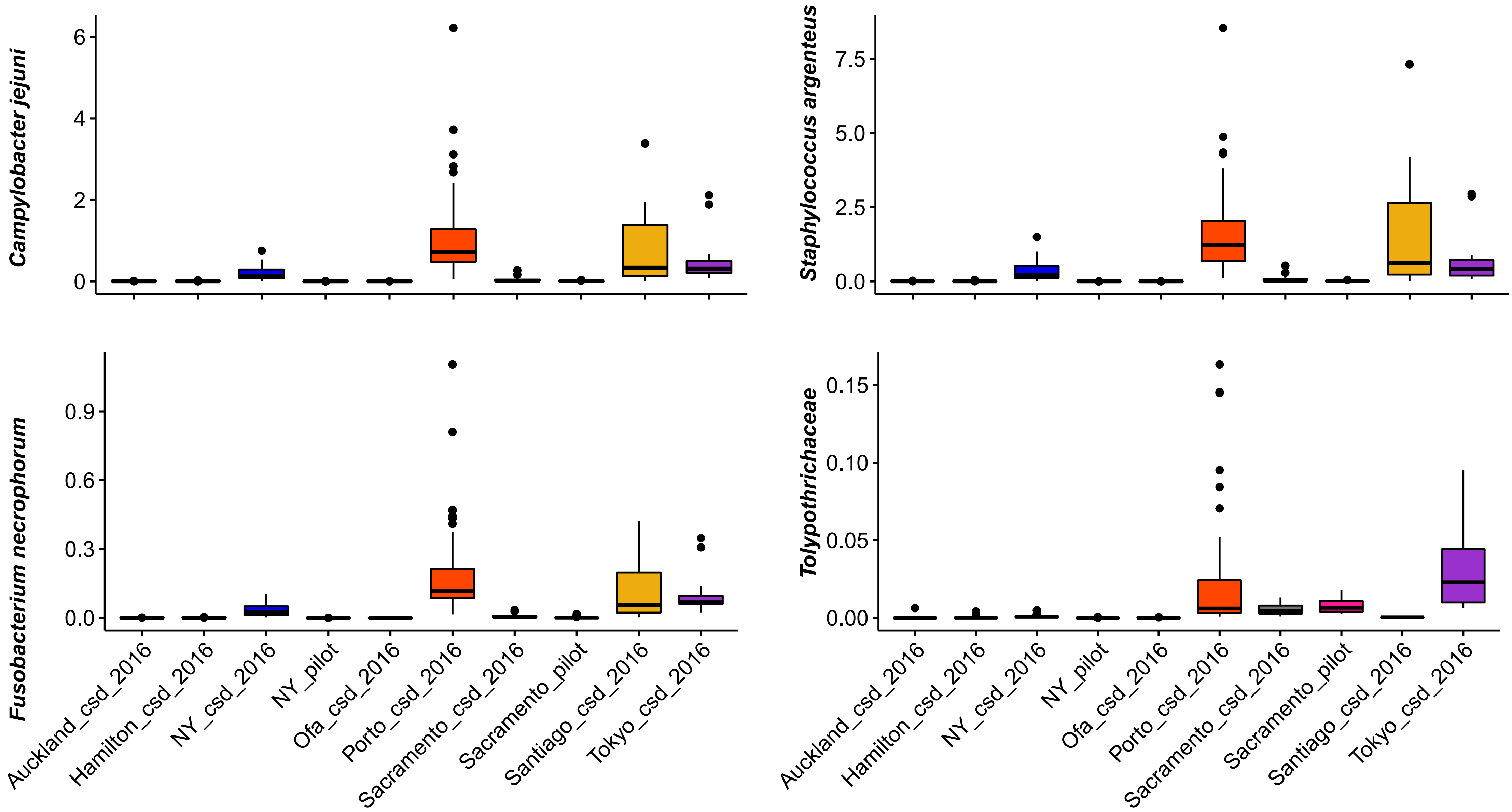

Campylobacter jejuni
0
2
4
6
Staphylococcus argenteus
0.0
2.5
5.0
7.5
Fusobacterium necrophorum
0.0
0.3
0.6
0.9
Tolypothrichaceae
0.00
0.05
0.10
0.15
Auckland_csd_2016
Hamilton_csd_2016
NY_csd_2016
NY_pilot
Ofa_csd_2016
Porto_csd_2016
Sacramento_csd_2016
Sacramento_pilot
Santiago_csd_2016
Tokyo_csd_2016
