# Supplementary figures and images for: Application of machine learning techniques for creating urban microbial fingerprints
Source: Biol Direct. 2019 Aug 16;14:13. doi: 10.1186/s13062-019-0245-x (PMC6697990; doi:10.1186/s13062-019-0245-x)

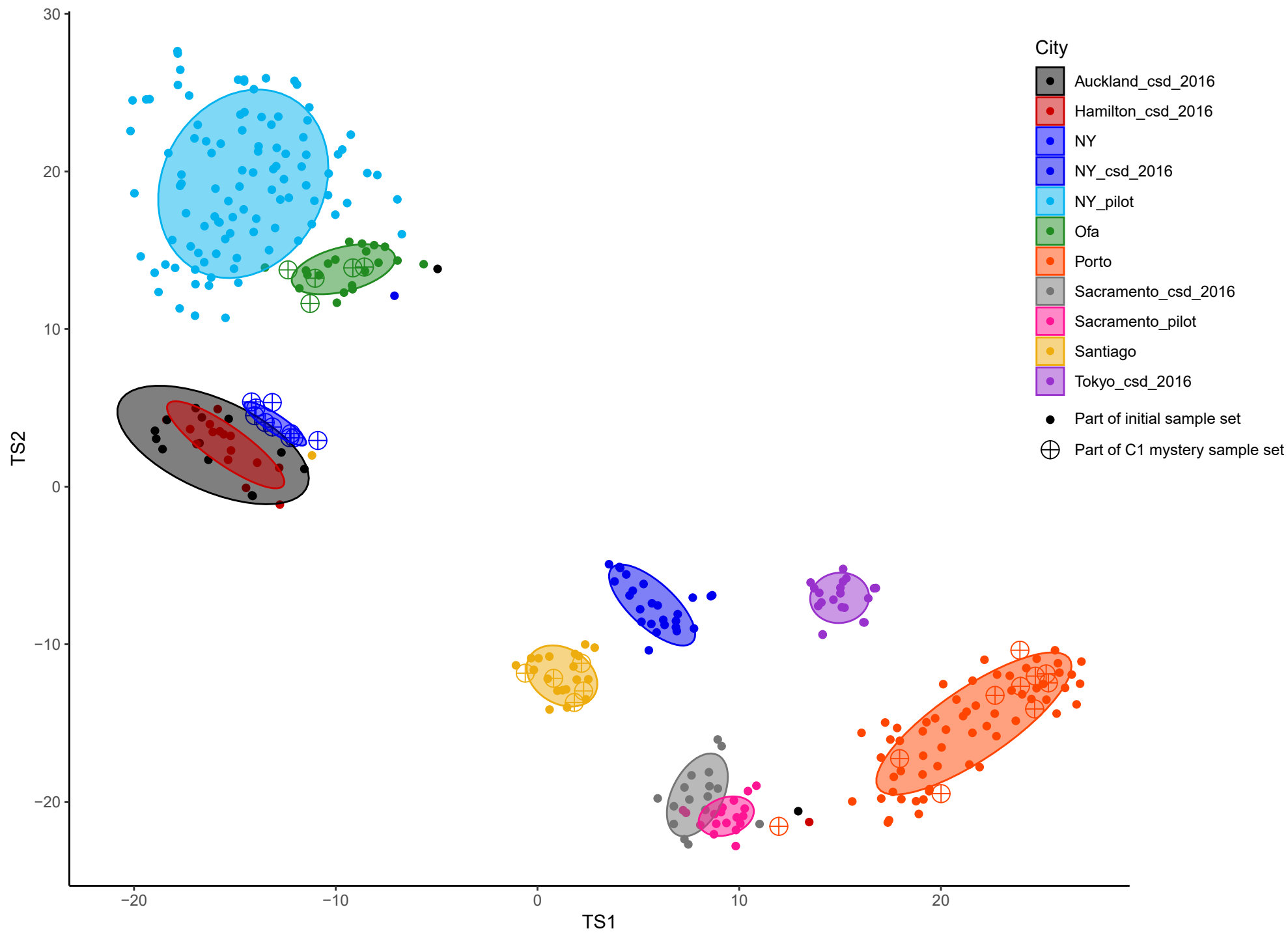

Supplement: Supplementary file 2 — Figure S2. t-SNE output to represent microbial profiles on two dimensions. Spearman dissimilarities were calculated from a set of 2347 taxonomic features which represent those present in at least 5% of samples with a minimum relative abundance of 0.1% in a single sample. Confidence regions are 70% confidence regions showing surface type. Size and shape of points indicates those which were part of the initial 311 sample set or those which were unlabeled. Information about city of origin was not used to generate these data and thus this highlights the ability to cluster samples by city of origin. (PDF 121 kb) [file 13062_2019_245_MOESM2_ESM.pdf]

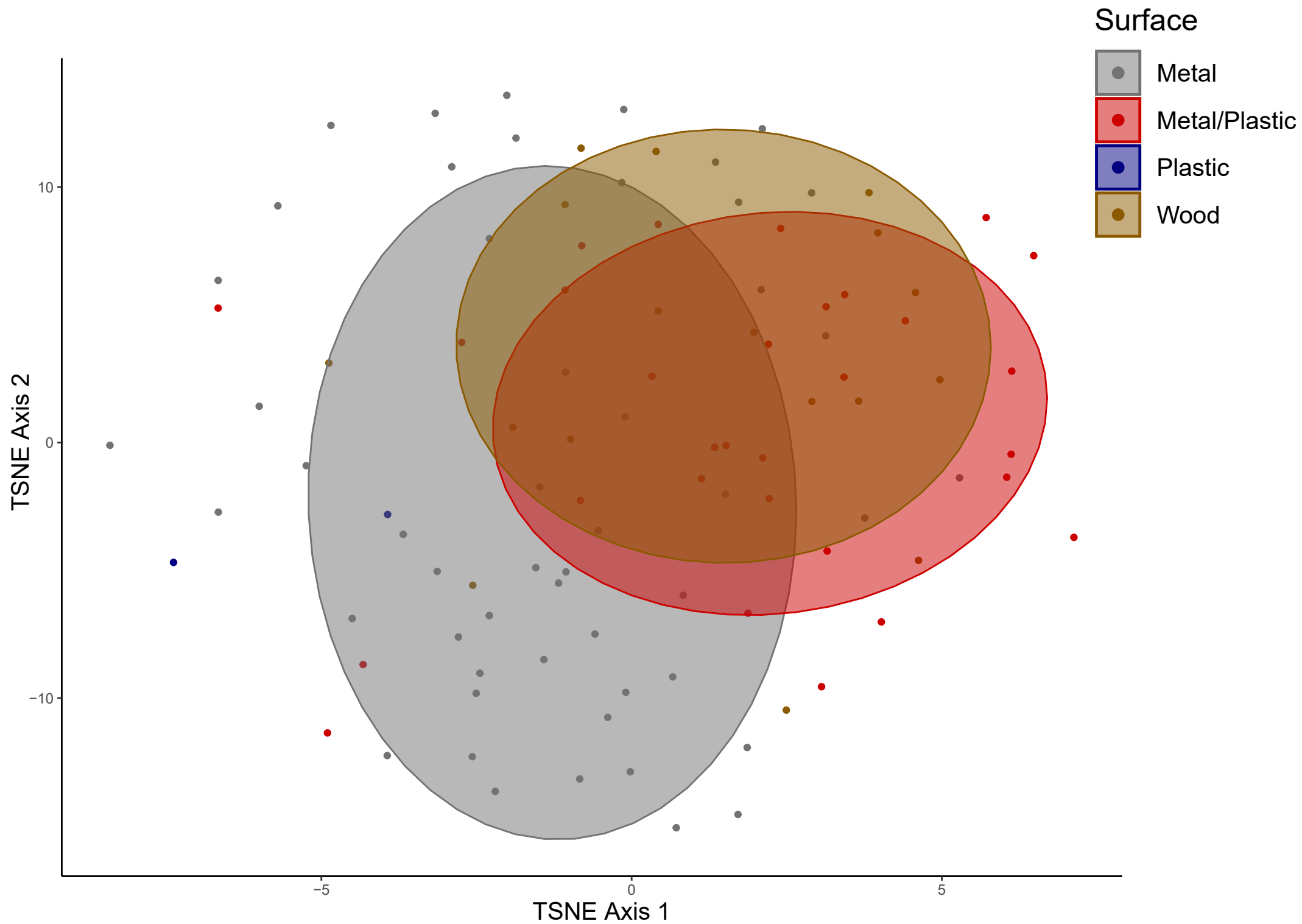

Supplement: Supplementary file 3 — Figure S3. t-SNE output to represent microbial profiles on two dimensions. Spearman dissimilarities were calculated from a set of 2239 taxonomic features which represent those present in at least 5% of samples with a minimum relative abundance of 0.1% in a single sample. Confidence regions are 70% confidence regions showing surface type. (PDF 85 kb) [file 13062_2019_245_MOESM3_ESM.pdf]
